# Rheumatoid arthritis increases the risk of heart failure-current evidence from genome-wide association studies

**DOI:** 10.3389/fendo.2023.1154271

**Published:** 2023-05-23

**Authors:** Min Wang, Kun Mei, Ce Chao, Dongmei Di, Yongxiang Qian, Bin Wang, Xiaoying Zhang

**Affiliations:** Department of Cardiothoracic Surgery, The Third Affiliated Hospital of Soochow University, Changzhou, Jiangsu, China

**Keywords:** rheumatoid arthritis, autoimmune disease, heart failure, NT-proBNP, Mendelian randomization analysis, genome-wide association study

## Abstract

**Background:**

Numerous studies have demonstrated that rheumatoid arthritis (RA) is related to increased incidence of heart failure (HF), but the underlying association remains unclear. In this study, the potential association of RA and HF was clarified using Mendelian randomization analysis.

**Methods:**

Genetic tools for RA, HF, autoimmune disease (AD), and NT-proBNP were acquired from genome-wide studies without population overlap. The inverse variance weighting method was employed for MR analysis. Meanwhile, the results were verified in terms of reliability by using a series of analyses and assessments.

**Results:**

According to MR analysis, its genetic susceptibility to RA may lead to increased risk of heart failure (OR=1.02226, 95%CI [1.005495-1.039304], *P*=0.009067), but RA was not associated with NT-proBNP. In addition, RA was a type of AD, and the genetic susceptibility of AD had a close relation to increased risk of heart failure (OR=1.045157, 95%CI [1.010249-1.081272], *P*=0.010825), while AD was not associated with NT-proBNP. In addition, the MR Steiger test revealed that RA was causal for HF and not the opposite (P = 0.000).

**Conclusion:**

The causal role of RA in HF was explored to recognize the underlying mechanisms of RA and facilitate comprehensive HF evaluation and treatment of RA.

## Introduction

Rheumatoid arthritis (RA) is an autoimmune disease with a worldwide lifetime prevalence of 1% (1), and more common in women, which accounts for 75% of all RA cases (2). RA is typically indicated by the presence of autoantibodies, including anti-cyclic citrullinated peptide and rheumatoid factor, years before the disease can be detected (3), and the most common clinical manifestations caused by these autoantibodies are distal joint pain and joint deformity caused by involvement of synovial joints. Current therapies for RA include antirheumatic drugs (DMARDs), anti-tumor necrosis factor-alpha inhibitors (e.g., adalimumab, etanercept, and infliximab) and non-tumor necrosis factor inhibitors (e.g., ababtreotide, rituximab, toximab) (4). If untreated or poorly controlled, it may lead to interrupted physical function and increased mortality owing to increased cardiovascular risk.

Despite progress in the treatment of RA, which achieves disease activity control in most patients, the life expectancy of RA patients remains low due to the complications of cardiovascular diseases (5, 6). It was found that RA patients had a risk of heart failure 1.87% higher than that of the general population (7), and it was not associated with cardiovascular risk factors (8). The incidence of sudden cardiac death of RA patients is twice that of normal controls, and it is secondary to non-ischemic heart disease, ischemic heart disease and arrhythmia (9). Meanwhile, it is shown that the prevalence of non-ischemic heart disease (heart failure) in RA patients is significantly higher than that of ischemic heart disease (10). N-Terminal Pro-Brain Natriuretic Peptide (NT-proBNP) is now established for the diagnosis of heart failure, but new evidence also points to the role of NT-proBNP in diagnosing myocardial ischemia in asymptomatic patients for primary prevention. NT-proBNP has been shown to be elevated in RA, and this elevation is not significantly related to cardiac function (11). Whether RA can directly affect the change of NT-proBNP, the causal relationship remains unknown. It is noteworthy that these observational studies have different sample size and the results are indeed dependent on confounding factors, and the specific mechanism has yet to be clarified.

Confirmation of causality is challenging due to complex confounders of RA and HF risk. The causal relationship of exposure and outcomes without bias was assessed, and the instrumental variables (IVs) were genetic variation in MR analysis (12). In virtue of the unique advantages of IVs, MR analysis is independent from conventional confounding factors, allowing causal inference (13, 14). Genome-wide association studies (GWAS) provide reliable IVs. In this study, MR analysis was performed on two samples to clarify the potential causality of HF risk and genetic susceptibility to RA and AD without interference from side effects of drug or common risk factors, which is critical for prevention and treatment of RA and even AD.

## Methods

### Study design and data sources

A two-sample MR approach and classical MR analysis were involved in this study. The data related to RA were acquired from a meta-analysis of GWAS, which included 14,361 cases and 42,923 controls. GWAS data for AD (42,202 cases and 17,6590 controls) were acquired online (https://www.finngen.fi/en). For the outcome dataset, single nucleotide polymorphisms (SNPs) for HF were acquired from a meta-analysis of GWAS (47,309 cases and 930,014 controls). The data for NT-proBNP were acquired from GWAS (21,758 samples). **Table 1** summarizes demographic profiles involved. The details of the GWAS are provided in **Supplementary Table 1**.

**Table 1:** Instrumental variable assessment and data source.

Traits	Data sources	Sample size (cases/controls)	Ancestry	R^2^(%) for RA/AD (Total)	F for RA/AD (Total)
Exposures					
RA	PMID:24390342	14,361/42,923	European		
AD	FinnGen	42,202/17,6590	European		
Outcomes					
HF	PMID:31919418	47,309/930,014	European	0.76/0.008	1442/44
NT-proBNP	PMID:33067605	21,758	European	0.76/0.008	1453/38

F=R^2^(N-K-1)/[K(1-R^2^)], R^2 = ^2×(1-EAF)×EAF×(b/SD)^2^, among which SD=SE×N^1/2^, where N refers to the sample size of GWAS, b refers to an effect estimated on adipokines, SE refers to the SD of b, and EAF refers to an effect allele frequency.

**Figure 1:**
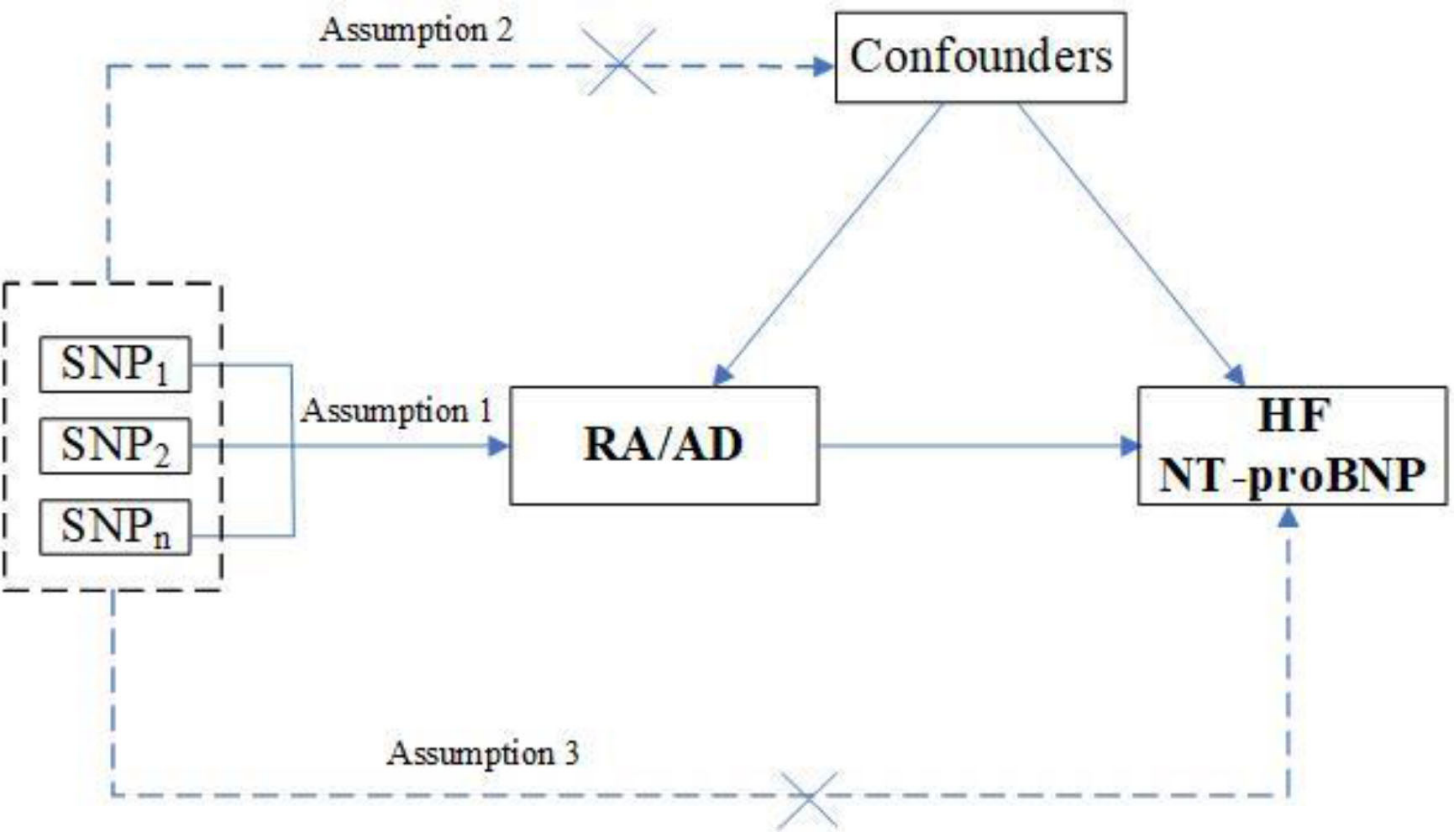
Study design flowchart of the Mendelian randomization study. The Mendelian randomization method is based on three hypotheses: 1. the instrumental variables is closely related to exposure; 2. instrumental variables is independent of any confounding factor; 3. instrumental variables affects the results only through exposure but not through other ways.

We performed a two-sample MR study to assess the causality of CVD risk and genetic susceptibility to RA. Herein, SNPs served as IVs (15). An overview of the research design is presented in **Figure 1**. The entire process satisfied the three main hypotheses of classical MR analysis: 1. exposure is directly affected IVs; 2. IVs had no correlation with confounders; 3. IVs directly impact outcome risk via exposure, instead of other pathways. Additionally, ethical approval was available for all original studies, along with informed consent. Herein, we followed the latest (STROBE-MR) guidelines (16).

### Ethical approval

A MR study by using GWAS summary statistics was employed in this study, and ethical approval had been obtained for each GWAS. The summary statistics were obtained online (https://www.ebi.ac.uk). All data are accessible and no restriction was set.

### Selection of IVs

Genetic variants that are closely related to RA (*P* < 5 ×10^−8^) were regarded as instrumental variables. We made sure to include only SNPs that were independent (r2<0.001 in 10.000kb) performing LD-clumping with a European reference panel from 1000G (17). Meanwhile, secondary phenotypes were searched for each SNP in order to exclude potential pleiotropic effects. We did not find SNPs associated with confounders (hypertension, diabetes, obesity, and smoking) in PhenoScanner V2. Specifically, SNPs corresponding to the outcome-related phenotypes (P < 5 ×10^−8^) were excluded, while other SNPs were kept. After that, variance (R^2^) and F-statistics were employed to evaluate the strength of instrumental variables so that weak-tool bias can be avoided (18). Herein, the formula is as follows: F=R^2^(NK-1)/[K(1-R^2^)], where N denotes the sample number of the chosen GWAS, K denotes the number of SNPs involved, and R^2^ denotes the explained variance (cumulative) of the chosen SNPs during exposure. F>10 indicates a strong correlation of exposure and instrumental variables, and the MR analysis results are independent on weak-tool bias.

### MR-analysis

All statistical analyses were conducted using R software (version 4.2.0, R Foundation for Statistical Computing), the MR analysis was performed using the “TwoSampleMR” package (version 0.5.6). For each set of IVs, we harmonized exposure and outcome data to ensure the effect sizes for each GWAS were aligned to the same alleles. Similarly, different exposures (e.g., AD) and outcomes (NT-proBNP) were adjusted in a similar way. The inverse variance weighting (IVW) method was dominant in the MR analysis (15). Meanwhile, MR-PRESSO, MR-RAPS, maximum-likelihood, MR-Egger, and median weighting were employed to clarify the causality (18). Different hypotheses about the effectiveness of IVs were made by using each method. Estimation of median weighting is executed if half of IVs are invalid. MR-Egger was used because it corrects for horizontal pleiotropy, despite lower statistical capability. Specifically, the MR-RAPS was responsible for horizontal multiplicity correction by contour scores adjusted, resulting in reduced deviation due to horizontal multiplicity. And the MR-PRESO method could automatically identify and remove outliers (IVW linear regression) to correct the MR estimation (19). The directionality that exposure causes outcome was verified using the MR Steiger test, P < 0.05 was regarded as statistically significant. These methods were used to comprehensively investigate causality.

### Multivariable Mendelian randomization analysis

Multivariable MR (MVMR) analysis was implemented for significant exposure-outcome pairs identified by univariate MR analysis. Specifically, four confounders, Diabetes (IEU GWAS ID: “ukb-b-10753”), Obesity (IEU GWAS ID: “finn-b-E4_OBESITY”), Hypertension (IEU GWAS ID: “finn-b-I9_HYPTENS”) and Smoking (IEU GWAS ID: “ieu-b-142”), were included for MVMR analysis. After combining the GWAS summary level datasets of exposure and the four confounders, it should be ensured that each IV is strongly correlated (P < 5e−8) with at least one or more of the exposure or the three confounders. Then, the SNPs within a window size of 10,000 kb were pruned under the threshold of r^2^ < 0.001 to mitigate LD. Finally, after excluding palindromic SNPs, outcome-related SNPs (P<0.05), and SNPs not present in outcome GWAS summary data, we used the IVW method to assess causal effects after adjusting for confounders.

### Pleiotropy and heterogeneity analyses

As primary analysis we applied the Causal Analysis Using Summary Effect Estimates (CAUSE) approach, which has been demonstrated to outperform other established methods to detect causal relationships in the presence of pleiotropy, CAUSE avoids more false positives induced by correlated horizontal pleiotropy than other methods (20). In this case, CAUSE analysis was conducted to determine whether the relationship between RA and HF was causal (causal model) or induced by correlated horizontal pleiotropy (shared model). When P<0.05 it means that the causal model is preferred over the shared one, indicated that the causal relationship between RA and HF is real and not a false positive due to the correlated horizontal pleiotropy. A series of methods were used for sensitivity analysis in this study. First, the heterogeneity of different SNP estimates was evaluated by the Cochran’s Q test. If *P* > 0.05, no heterogeneity was indicated. Although the random-effects model could be used, the fixed-effect IVW method was dominant. Second, the horizontal pleiotropy of IVs was investigated by using the MR-Egger intercept method (21). Average of the horizontal pleiotropic effect was estimated based on the intercept across SNPs in the MR-Egger test, and the IVW estimate might be biased if *P* < 0.05. Third, a single SNP could generate the results was verified by using the leave-one-out sensitivity test. Leave-one-out method shown how the IVW causal effect when remove each variant from the analysis. This allows to detect heterogeneity since if the IVW changes drastically, that means that a variant is contributing way more than the others. Importantly, this is not always a sign of pleiotropy, but always a sign of heterogeneity in the data being analyzed. Fourth, the presence of pleiotropy was directly detected by generating funnel and forest plots. “Two-Sample MR”, “MR-PRESSO”, “CAUSE” and “mr.raps” packages in R software were used for statistical analysis.

## Results

### Causality of genetic susceptibility to RA and AD on the risk for HF

As shown in **Table 2**, results obtained by the IVW method indicated that RA was related to increased risk of HF. As observed, the prevalence of HF in RA cases was 1.014-fold that of the control group (95% CI [1.0009-1.0281], OR=1.014, *P*=0.036) (**Supplementary Figure 1**), and increase of the OR of AD by one unit leads to increased HF risk (95% CI [1.010-1.081], OR=1.045, *P*=0.011) (**Supplementary Figure 3**). MR analysis of RA and HF indicated that the results of the Weighted median analyses were highly consistent with those obtained by the IVW method. In the strict CAUSE, the causal model was shown to be a better fit than the sharing model (95% CI [2.461-2.823], OR=2.642, p = 1.8e-30), indicating a causal association between RA and HF. More supporting statistics were listed in **Supplementary Table 2**. MR analysis of AD and HF showed that the results of the MR Egger analyses were highly consistent with those obtained by the IVW method. The causal assumption of RA or AD and HF was verified via the MR Steiger test, and the result showed RA or AD influence on HF was the correct causal direction (P = 0.000). The details of the MR Steiger test are provided in **Supplementary Table 3**.

**Table 2:** MR estimates of RA and AD on the risk for HF.

Disease	Methods	SNPs(n)	OR	95%CI	*P*-value
RA	MR Egger	112	1.006100	0.983752-1.028956	0.596722
	Weighted median	112	1.006559	0.983870-1.029771	0.574114
	IVW	112	1.014421	1.000941-1.028084	0.035929
	Simple mode	112	1.060100	1.012280-1.110178	0.014707
	Weighted mode	112	1.009812	0.988696-1.031380	0.367098
AD	MR Egger	39	1.074839	1.014617-1.138636	0.01899
	Weighted median	39	1.032231	0.992336-1.07373	0.114699
	IVW	39	1.045157	1.010249-1.081272	0.010825
	Simple mode	39	0.982959	0.904143-1.068645	0.689153
	Weighted mode	39	1.037358	1.000383-1.075699	0.054899

### Causality of the risk for NT-proBNP and genetic susceptibility to RA and AD

As shown in **Table 3**, the prevalence of NT-proBNP (β=-0.0114, SE =0.0150, *P*=0.4467) in the RA group was not significantly different from that of the control group (**Supplementary Figure 2**). The results listed were consistent with those obtained by the IVW method. Meanwhile, no significant association was observed between AD and NT-proBNP risk (β=0.0722, SE =0.0265, *P*=0.7851) (**Supplementary Figure 4**). It was also confirmed by analyses listed in the table.

**Table 3:** MR estimates of RA and AD on the risk for NT-proBNP.

Disease	Methods	SNPs(n)	β	SE	*P*-value
RA	IVW	114	-0.011421	0.015009	0.446705
	Weighted median	114	0.014745	0.026135	0.572620
	MR Egger	114	-0.014908	0.027667	0.591073
	Weighted mode	114	0.009568	0.026682	0.720572
	Simple mode	114	0.0176783	0.052438	0.736644
AD	IVW	52	0.007217	0.026466	0.785090
	Weighted median	52	0.037362	0.037515	0.276857
	MR Egger	52	0.041910	0.045491	0.361601
	Weighted mode	52	0.0373620	0.036093	0.305781
	Simple mode	52	-0.041060	0.589407	0.589407

### Results of multivariable Mendelian randomization analysis

As shown in **Table 4**, We performed an MVMR analysis to assess the causal effect of RA on HF after adjusting for four confounding factors (diabetes, obesity, hypertension and smoking). MVMR analysis identified that all of these four confounders were taken into account, the causal relationship between RA and HF was not obvious (OR = 1.022968, 95% CI [0.9994881-1.047000], P = 0.055266). indicating that no significant direct causal effect was detected for RA on HF risk, while jointly modeling diabetes, obesity, hypertension and smoking.

**Table 4:** MVMR analysis for assessing the causal effect of RA on HF.

Exposure	SNPs	OR	95% CI	P-value	F-statistic
RA	38	1.022968	0.9994881-1.047000	5.526636e-02	37.79063
Diabetes mellitus	37	1.657085	0.7339741-3.741182	2.241354e-01	3.369213
Obesity	2	1.062153	0.9828610-1.147841	1.276904e-01	10.22393
Hypertension	28	1.190533	1.1231576-1.261950	4.423231e-09	15.66378
Smoking	17	1.133324	1.0477772-1.225855	1.774866e-03	25.55431

### Analysis of horizontal pleiotropy and heterogeneity

As shown in **Table 5**, a series of methods were employed for MR analysis regarding the correlation of RA, AD and HF to determine the presence of significant horizontal pleiotropy and heterogeneity in the present study. First, the *P*-value was > 0.05 in the heterogeneity test, demonstrating that SNPs had negligible heterogeneity (**Table 5**). The fixed-effect IVW method was dominant in this MR analysis. The “leave-one-out” sensitivity analysis demonstrated that IVs involved in the present study had negligible impact on such results (**Supplementary Figures 5**-**8**), and the funnel plot illustrates an asymmetric distribution of single IVs (**Supplementary Figure 9**), suggesting that the causality was not likely to be affected by potential bias. The MR Steiger test indicated that there was no reverse causality (**Supplementary Table 3**).

**Table 5:** Heterogeneity and pleiotropy test of RA and AD from HF and NT-proBNP GWAS.

Exposure	Outcomes	Pleiotropy test			Heterogeneity test					
		MR-Egger			MR-Egger			Inverse-variance weighted		
		Intercept	SE	*P*	Q	Q_-_df	Q_-_ *p*val	Q	Q_-_df	Q_-_ *p*val
RA										
	HF	0.001384	0.001547	0.372925	109.7300	110	0.489326	110.530	111	0.494726
	NT-proBNP	0.000523	0.003477	0.880793	117.3168	112	0.346731	117.3404	113	0.370953
AD										
	HF	-0.0039	0.0033	0.2475	60.5797	37	0.0086	62.8402	38	0.0068
	NT-proBNP	-0.0046	0.0049	0.3532	45.4325	47	0.5376	46.3118	48	0.5422

## Discussion

In the present study, MR analysis was first performed to investigate the potential causal relationship of HF risk and the susceptibility to RA. RA is the most common autoimmune disease. The causal relationship of HF risk and AD was thus evaluated by MR analysis. The results showed that the genetic susceptibility to RA and AD was correlated with an increase in HF risk. The MR Steiger test further showed that there was no evidence of reverse causality in our study. The limited evidence from MR analysis supported the potential causal relationship between RA and AD and HF risk.

HF is a cardiovascular syndrome associated with RA and also contributes to the incidence and death of RA (22). In the population-based RA cohort, the incidence of HF was about twice the incidence in the general population (22, 23). As a complex clinical syndrome, HF involves a variety of potential risk factors and causes, among which hypertension and ischemic heart disease are most common (24). Clinically, HF is classified based on the left ventricular ejection fraction (LVEF): 1. Reduced LVEF is defined as ≤40%, i.e. those with a significant reduction in LV systolic function. This is designated as HFrEF. 2. Patients with a LVEF between 41% and 49% have mildly reduced LV systolic function, i.e. HFmrEF. 3. Those with symptoms and signs of HF, with evidence of structural and/or functional cardiac abnormalities and/or raised natriuretic peptides (NPs), and with an LVEF ≥50%, have HFpEF (25). Along with aggravated population aging, the prevalence of HFpEF has been rising in recent years. A recent retrospective study found that 64% of the RA patients are combined with HFpEF (26). HFpEF is more common among RA patients compared to the general HF population without RA (27). A follow-up survey using cardiac ultrasonography showed that the development of subclinical changes in the diastolic function among RA patients was more rapid within 5 years compared to the general population (28). Mantel et al. compared the incidence of 10,000 Swedish patients with ischemic and non-ischemic heart failure. They reported a rapid increase in the HF risk following the onset of FA and a close connection with high disease activity (10). RA patients were related to a higher incidence of HF and IHD throughout the course of observation, and RA was more significantly correlated with the high HF risk (29). Recent advances in the treatment of RA have decreased the incidence of cardiovascular diseases in RA patients, but these patients are still at a higher risk for IHD. Besides, the HF risk increases as the duration and severity of RA increase (10, 30). Nicola et al. proved that compared to the non-RA population, the risk of congestive heart failure was significantly increased in the RA population, with an odds ratio of 1.87 during the 30-year follow-up (22). Similarly, according to Wolfe and Michaud, HF was common among RA patients (22). Michael J Ahlers et al. performed a retrospective case-control study of 9,889 RA patients and 9,889 controls without autoimmune diseases, who were matched for age, gender, and race. It was found that the HF risk was increased by 21% in RA patients and such an increase was irrelevant to the conventional cardiovascular risk factors (26). This estimate agrees with the increased HF risk associated with RA at the Swedish and Danish National Patient Registry (10, 29). Nevertheless, the above reported increase in the HF risk was smaller than that reported by Nicola in the presence of RA, which was 87% (22). Recently, some scholars reported that among RA patients diagnosed in Denmark from 1978 to 2008, RA was associated with an increase in HF-related admissions (31). The above evidence has indicated that RA does increase the risk of HF. Four main factors have been identified as contributors of a higher HF risk in RA patients (32): 1. Conventional cardiovascular risk factors, including smoking, dyslipidemia, hypertension, obesity and diabetes, which usually exist concurrently with the risk factors for RA; 2. The use of glucocorticoids and non-steroidal anti-inflammatory drugs will increase the HF risk; 3. The presence of anti-citrulline peptide antibodies and rheumatoid factors in RA patients was an independent risk factor for HF; 4. An increase in the RA disease activity alongside a continuous cardiovascular impact of systemic inflammation is another primary risk factor for HF.

However, the increased prevalence of hypertension and IHD in RA patients may not fully explain the higher HF risk in RA patients (24). A previous study showed that a significant increase in the mortality of HF among RA patients might be related to coronary artery disease (CAD) (22). Other research showed that RA is a typical chronic inflammatory disease and related to an increase in the HF risk. The latter, however, is uncorrelated with the conventional cardiovascular risk factors (including CAD) (10, 29, 33). The HF phenotype in RA patients is different from that in non-RA patients. The former usually presents with diastolic dysfunction, hypotension and high ejection fraction. Thus, RA and non-RA patients may vary in the mechanism of myocardial injury (27, 34). The newly diagnosed RA patients were associated with a significant increase in the incidence of HF events five years before the diagnosis, although few of them presented with typical features of cardiovascular risks, including hypertension and hypercholesterolemia. These facts suggest that CVD is not only a late complication of RA (35). RA-related inflammation may be a critical factor for the progression to HF. The HF risk may be even increased in an absence of IHD risk if the patients have RA-related inflammation. It has been reported that the risk of non-ischemic heart failure is increased at an early stage and closely connected with the severity of RA (10). In another study, the SLE/RA inpatients were analyzed, and the prevalence of HF in the population was 16.4%. Besides, the likelihood of HF in RA patients was significantly lower than that in SLE (36). The above results proved from another perspective that RA-related HF is not caused by shared risk factors alone, since SLE and HF also share some common risk factors. PARK E et al. found that an increase in HF risk in RA patients might not be explained by IHD alone. Non-ischemic HF is related to the severity of RA, implying that RA-related factors and autoimmune process are related to the risk of the HF phenotype above (37).

In the present study, the causal relationship between RA and NT-proBNP was analyzed, but the result was negative. Recently, Baniaamam et al. conducted a prospective study of 51 RA patients, where echocardiography and baseline tests were performed on those with moderate to high disease activity, along with an assessment after six months of treatment with anti-tumor necrosis factor. Although the NT-proBNP level was decreased by 23% after six months of treatment, no adverse effect on the cardiac function was observed (38). The above results suggest that the RA-related impact on cardiac function is not manifested as changes in NT-proBNP. However, controversy continues over the predictive performance of HF-related biomarkers, such as B-type natriuretic peptide (BNP) or NT-proBNP, for cardiac injury. Some authors believe that these factors are sensitive, non-invasive predictors for subclinical CVD and are all-cause mortality predictors independent of conventional risk factors for CV (39). Evidence has shown that an increased NT-proBNP level in RA patients is related to inflammatory markers (40). However, some researchers did not prove the relationship between the NTproBNP level and left ventricular function in RA patients (41, 42), which also agreed with ours findings.

The relationship between RA and NT-proBNP is complex. In this study, there was no causal relationship between RA and serum NT-proBNP level. In the study of Armstrong et al. although researchers observed an increase in the median NT-proBNP level in the RA group, the increase in NT-proBNP level was significantly correlated with DAS28 and age, and had no direct correlation with RA itself (43). In addition, NT-proBNP may play an indispensable role in regulating the immune system and endocrine system (44–46), including the aging process of individuals, etc (47). These findings all reveal that NT-proBNP levels increase with age, so we speculate that the increased NT-proBNP levels in RA patients may be related to accelerated aging, rather than causally related to the disease itself. However, studies have shown that accelerated aging only explains 16% of the increase in BNP in RA patients (48). Therefore, the increase of BNP in RA patients is largely due to other unknown causes.

DMARDs and TNF-α inhibitors are usually prescribed as standard treatments for RA (42). TNF-α inhibitors are effective for controlling the activity and progression of RA. However, their risks in increasing incidence and deaths of cardiovascular diseases remain disputable, particularly RA patients already with a higher risk for cardiovascular complications (49). One study indicated that a higher dose of TNF-α inhibitors may cause HF deterioration and shortened life span (50). According to a randomized placebo-controlled clinical trial, TNF-α inhibitors did not have a considerable efficacy when used to treat symptomatic HF patients (51). Danish scholars performed a follow-up of RA patients that lasted for over 20 years, and it was found that the biological treatments for RA did not change the risks of IHD and HF (29). According to another study, the dose of glucocorticoids and TNF inhibitors was adjusted in the multivariate regression analysis, and it was found that the increased risk of HF in RA patients was independent of these drugs (31).

Inflammation is considered as a critical mechanism for the development of HF, especially HFpEF (52). Both ESR and CRP were correlated with increased risk of HF in RA patients (10). Evidence from the Mayo Clinic suggests that a higher level of inflammatory markers is related to a higher risk of HF (53). It has been found that an increase in the inflammatory activity related to the pathogenesis of RA may have myocardial effects, leading to HF shortly after RA diagnosis. In sepsis, TNF-α and other cytokines were related to the reduction in myocardial contractility after *in vitro* exposure for ≥10 min (54). Cardiomyocytes may also respond to inflammatory stimuli and express chemokines, cytokines, and cell adhesion molecules, leading to leukocyte recruitment and reduced cardiomyocyte contractility (55). Inflammation can also induce endothelial dysfunction, myocardial hypertrophy and fibrosis, which further results in HF (56). The incidence of HFpEF is also higher in other diseases related to chronic inflammation, such as obesity, diabetes and chronic kidney disease. It is implied that an increase in circulating proinflammatory cytokines in RA patients may be a critical factor in the pathogenesis of HF (57). Interestingly, those with the highest level of C-reactive protein (CRP) are also faced with the highest risk for HF, which highlights the role of inflammation in the pathogenesis. After stratified based on HF subtypes, the CRP level was higher in HFpEF than in HFrEF, indicating that inflammation might be a more important risk factor for HFpEF in RA (26).

RA is a chronic autoimmune inflammatory disease. Our study proved that RA was related to a higher risk for HF. To verify the results, MR analysis was performed, and a potential causal relationship of HF risk and the genetic susceptibility to AD was indicated. This finding coincided with our expectations. Another recent study showed that as an autoimmune disease, SLE was related to a higher risk of venous thromboembolism, ischemic cerebral infarction, and HF (58). Some researchers also performed MR analysis for this purpose, and it was found that RA was correlated with a higher risk of angina, hypertension, arrhythmia, and coronary heart disease (59). Others reported a correlation between MS and the risk of CAD, myocardial infarction, HF, and cerebral stroke (60). All the results above are consistent with our findings.

The clinical diagnosis and treatment of AD and HF should be carefully evaluated, considering the causal relationship of HF risk and the genetic susceptibility for RA and AD. In fact, rheumatologists have become increasingly aware of the relationship between CVD and RA. In the European Society of Cardiology guideline, RA is considered as an independent cardiovascular risk factor (61). The European League Against Rheumatism (EULAR) has published official advice for monitoring CV risk in RA patients (62). It is suggested that the CVD risk score should be multiplied by 1.5 in RA patients. Such a correction may improve the estimate of the cardiac risk in these patients. Therefore, earlier preventive tests and medication treatment are recommended if necessary.

### Advantages and limitation

A recent report involved MR analysis of the genetic susceptibility for cardiovascular risks. So far, the causal relationship between CVD risk and SLE and other autoimmune diseases has been analyzed, but few studies have been devoted to the potential relationship of HF risk and RA through MR analysis. We first performed MR analysis on RA and even AD and HF risk to identify any causal relationship. Secondly, large-scale GWAS was employed to collect more comprehensive genetic data in RA and HF, thereby avoiding the influence of conventional confounding factors and eliminating the potential of reverse causality. Lastly, consistent results were obtained through several repeat analyses, and an absence of biases was verified by the heterogeneity and pleiotropy analyses.

However, our study had some limitations. Firstly, pleiotropy was analyzed using multiple methods, but potential multiplicity might still exist. Secondly, we reported a lower OR value, compared with other studies, and more studies are needed to further document the clinical significance of this OR value. Thirdly, the F- statistics of obesity in MVMR analysis is lower than 10, which may cause a certain bias in the statistical results of MVMR, and the interpretation of the results should be very cautious.

## Summary

In conclusion, our study found the first evidence supporting the potential causal relationship of HF risk and RA and AD, which facilitates further investigation into the pathogenesis of RA and AD and comprehensive assessment of the RA-related HF and the associated treatments. Further studies are required to reduce the incidence and mortality of RA-related HF.

## Data availability statement

The original contributions presented in the study are included in the article/**Supplementary Material**. Further inquiries can be directed to the corresponding authors.

## Author contributions

MW and KM designed the study and drafted the article. CC and BW conducted data acquisition. DD, YQ and XZ performed data analysis and manuscript revision. All authors contributed to the article and approved the submitted version.

## Supplementary material

Supplementary Figure 1 Mendelian randomization analysis of RA and the risk of HF.

Supplementary Figure 2 Mendelian randomization analysis of RA and the risk of NT-proBNP.

Supplementary Figure 3 Mendelian randomization analysis of AD and the risk of HF.

Supplementary Figure 4 Mendelian randomization analysis of AD and the risk of NT-proBNP.

Supplementary Figure 5 The MR “leave-one-out” sensitivity analysis of RA on HF.

Supplementary Figure 6 The MR “leave-one-out” sensitivity analysis of RA on NT-proBNP.

Supplementary Figure 7 The MR “leave-one-out” sensitivity analysis of AD on HF.

Supplementary Figure 8 The MR “leave-one-out” sensitivity analysis of AD on NT-proBNP.

Supplementary Figure 9 Funnel plots of RA/AD with HF/BNP. The X-axis represents odds ratio (OR), and the Y-axis represents standard error (SE). (A)RA to HF. (B) AD to HF. (C) RA to NT-proBNP. (D)AD to NT-proBNP.

Supplementary Table 1 SNPs used to analyze the causal relationship between RA and HF, RA and NT-proBNP, AD and HF, AD and NT-proBNP.
