# Supplementary figures and images for: Rheumatoid arthritis increases the risk of heart failure-current evidence from genome-wide association studies
Source: Front Endocrinol (Lausanne). 2023 May 23;14:1154271. doi: 10.3389/fendo.2023.1154271 (PMC10242133; doi:10.3389/fendo.2023.1154271)

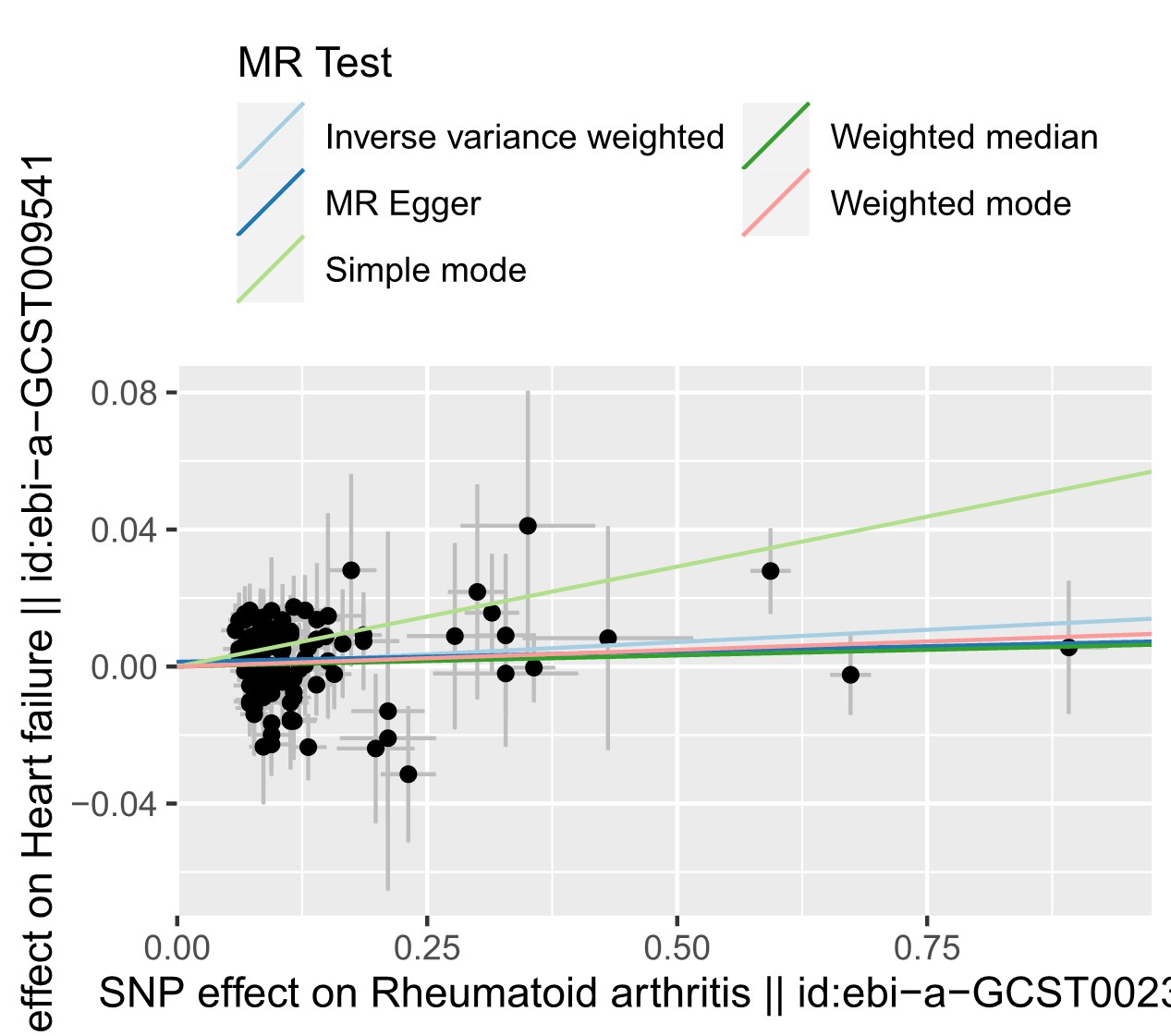

Supplement: Supplementary Figure 1 — Mendelian randomization analysis of RA and the risk of HF. [file Image_1.jpeg]

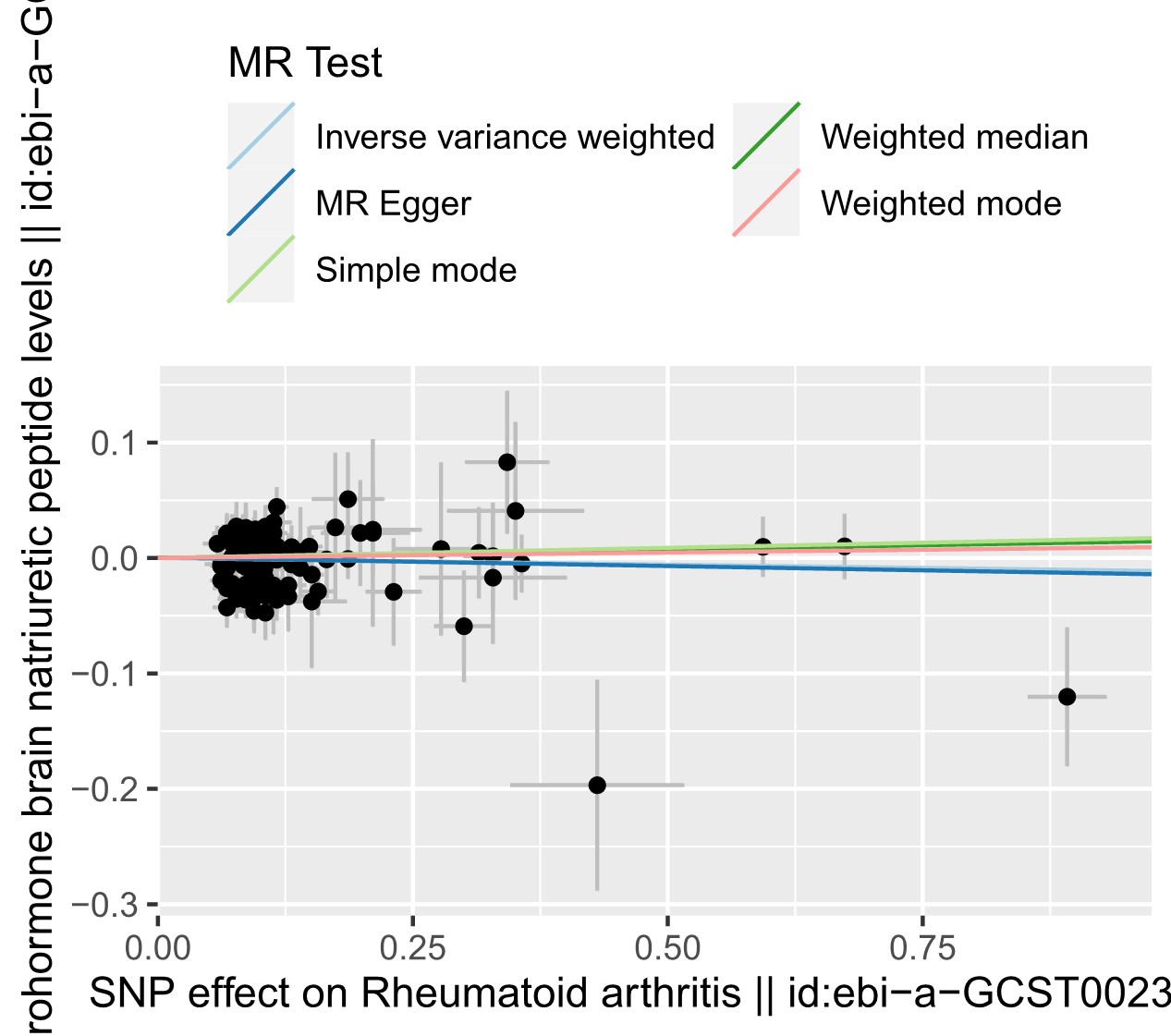

Supplement: Supplementary Figure 3 — Mendelian randomization analysis of AD and the risk of HF. [file Image_3.jpeg]

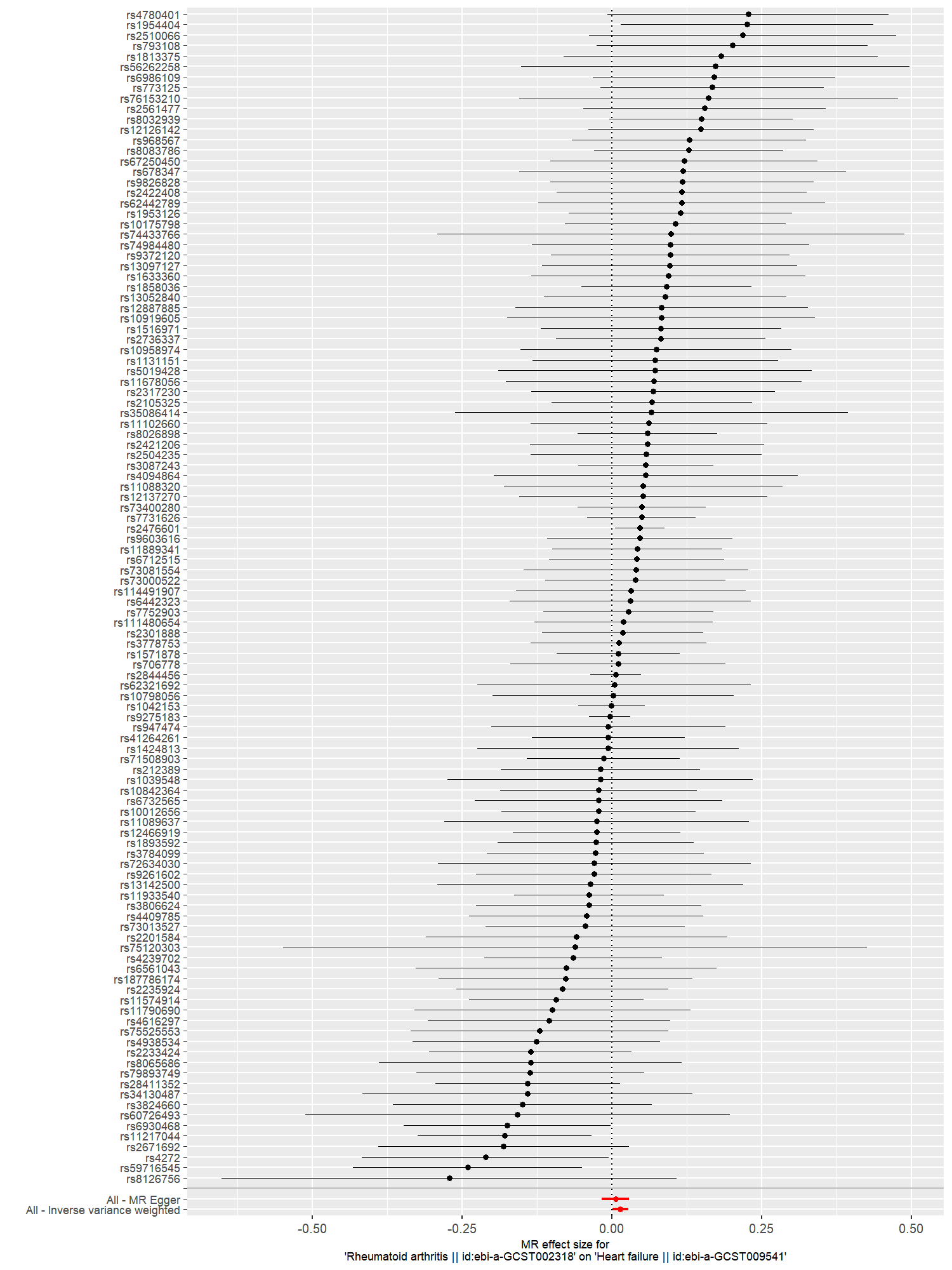

Supplement: Supplementary Figure 5 — The MR “leave-one-out” sensitivity analysis of RA on HF. [file Image_5.tiff]

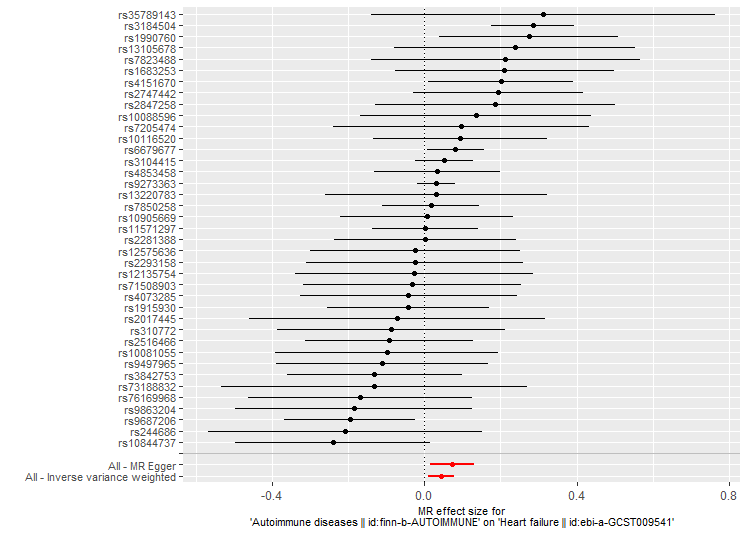

Supplement: Supplementary Figure 6 — The MR “leave-one-out” sensitivity analysis of RA on NT-proBNP. [file Image_6.png]

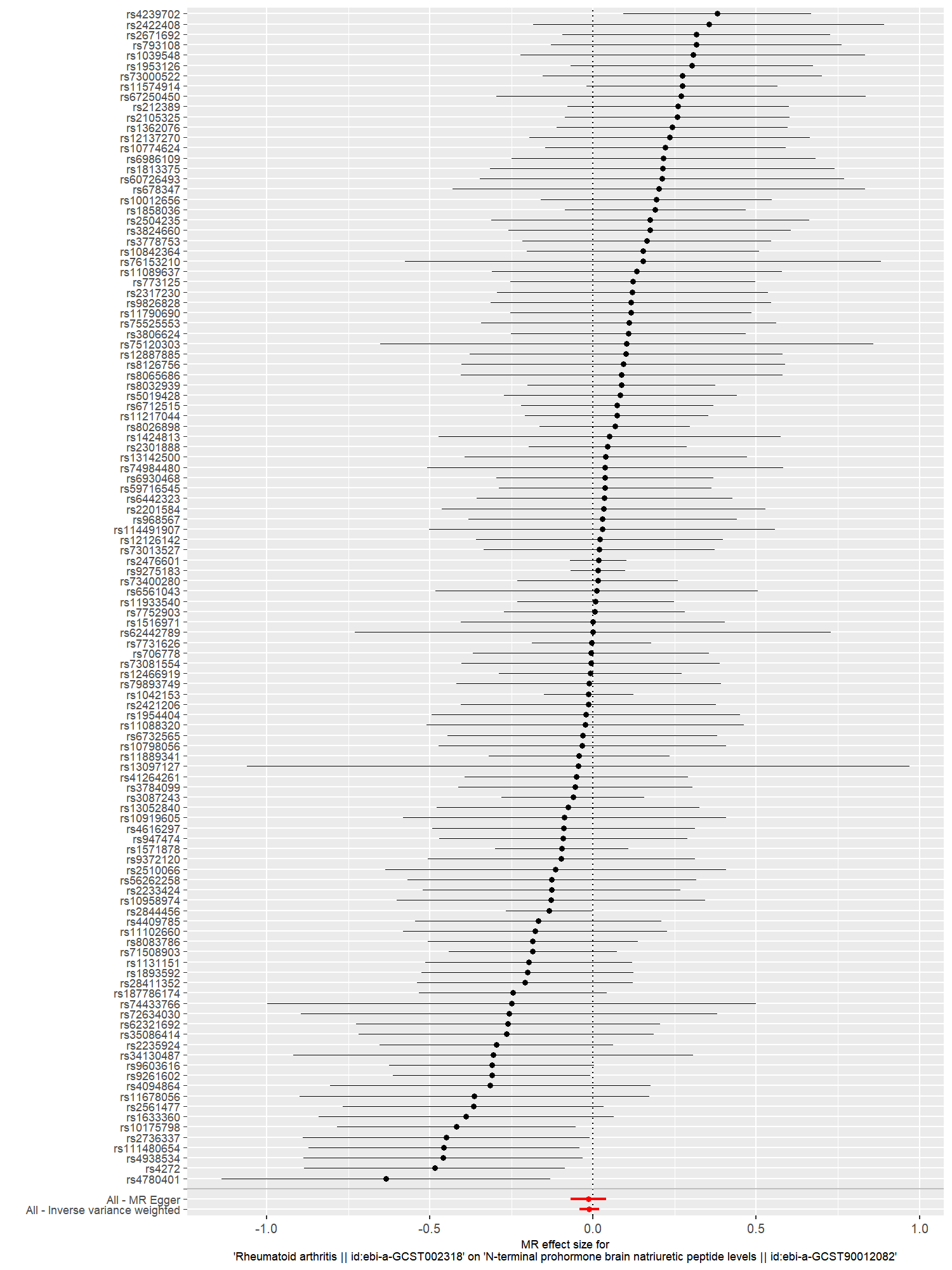

Supplement: Supplementary Figure 7 — The MR “leave-one-out” sensitivity analysis of AD on HF. [file Image_7.tiff]

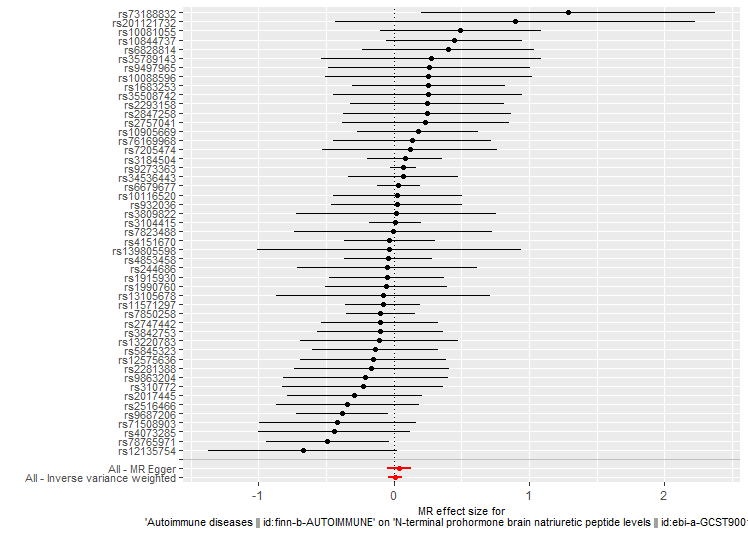

Supplement: Supplementary Figure 8 — The MR “leave-one-out” sensitivity analysis of AD on NT-proBNP. [file Image_8.png]

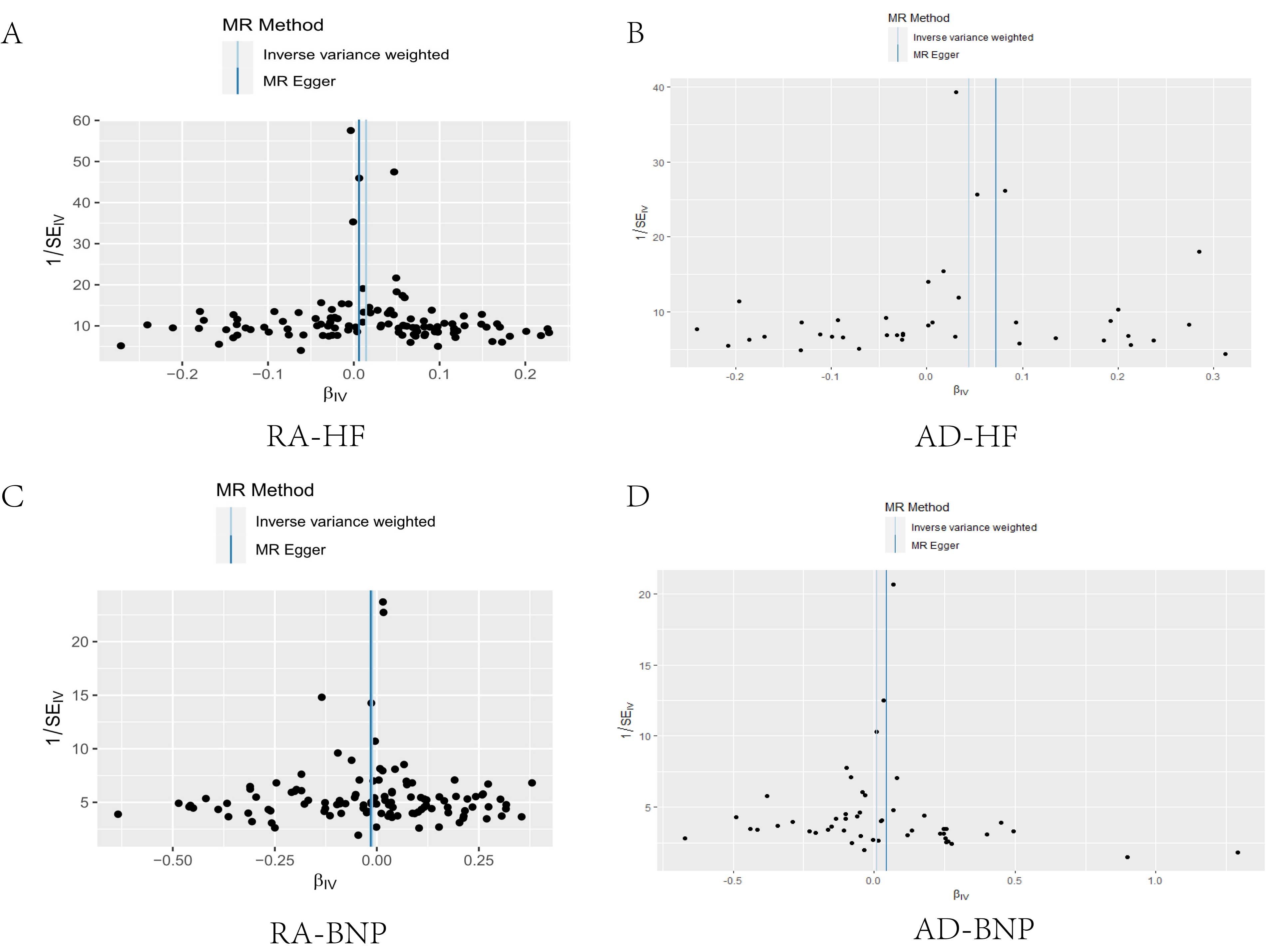

Supplement: Supplementary Figure 9 — Funnel plots of RA/AD with HF/BNP. The X-axis represents odds ratio (OR), and the Y-axis represents standard error (SE). (A)RA to HF. (B) AD to HF. (C) RA to NT-proBNP. (D)AD to NT-proBNP. [file Image_9.jpeg]
