# Supplementary material for: Rheumatoid arthritis increases the risk of heart failure-current evidence from genome-wide association studies
Source: Front Endocrinol (Lausanne). 2023 May 23;14:1154271. doi: 10.3389/fendo.2023.1154271 (PMC10242133; doi:10.3389/fendo.2023.1154271)
Supplement: Supplementary file 11 [file Table_2.docx]

**Supplementary Table 2. CAUSE analysis of RA and HF.**

| **Comparison** | **Model 1** | **Model 2** | **Delta_elpd** | **Se_delta_elpd** | **Z** |
| --- | --- | --- | --- | --- | --- |
| 1 | Null | Sharing | -42.242 | 4.253 | -9.933 |
| 2 | Null | Causal | -51.702 | 5.072 | -10.193 |
| 3 | Sharing | Causal | -9.460 | 0.829 | -11.414 |
