# Supplementary material for: Rheumatoid arthritis increases the risk of heart failure-current evidence from genome-wide association studies
Source: Front Endocrinol (Lausanne). 2023 May 23;14:1154271. doi: 10.3389/fendo.2023.1154271 (PMC10242133; doi:10.3389/fendo.2023.1154271)
Supplement: Supplementary file 12 [file Table_3.docx]

| Outcome | Exposure | correct causal direction | p value |
| --- | --- | --- | --- |
| HF | RA | 1 | 0.000 |
| HF | AD | 1 | 4.19991E-45 |
| NT-proBNP | RA | 1 | 0.000 |
| NT-proBNP | AD | 1 | 0.000 |

**Supplementary Table 3**. Summary results of the MR Steiger
